# The Elusive Path Toward Measuring Health Outcomes: Lessons Learned From a Pseudo-Randomized Controlled Trial of a Large-Scale Mobile Health Initiative

**DOI:** 10.2196/14668

**Published:** 2019-08-21

**Authors:** Patricia Mechael, Nadi Nina Kaonga, Subhashini Chandrasekharan, Muthu Perumal Prakash, Joanne Peter, Aakash Ganju, Nirmala Murthy

**Affiliations:** 1 HealthEnabled Washington, DC United States; 2 All of Us Research Program National Institutes of Health Bethesda, MD United States; 3 Tufts University School of Medicine Boston, MA United States; 4 Foundation for Research in Health Systems Chennai India; 5 Johnson & Johnson Cape Town South Africa; 6 Saati Health Mumbai India; 7 Foundation for Research in Health Systems Bengaluru India

**Keywords:** India, mHealth, maternal health, child health, research

## Abstract

Mobile health (mHealth) offers new opportunities to improve access to health services and health information. It also presents new challenges in evaluating its impact, particularly in linking the use of a technology intervention that aims to improve health behaviors with the health outcomes that are impacted by changed behaviors. The availability of data from a multitude of sources (paper-based and electronic) provides the conditions to facilitate making stronger connections between self-reported data and clinical outcomes. This commentary shares lessons and important considerations based on the experience of applying new research frameworks and incorporating maternal and child health records data into a pseudo-randomized controlled trial to evaluate the impact of mMitra, a stage-based voice messaging program to improve maternal, newborn, and child health outcomes in urban slums in India.

## Background

Over the past 10 years, there has been a rapid increase in the adoption and use of mobile technology by the health sector globally as a tool to increase access to health services and health information, strengthen health systems, improve the quality of care by health professionals, and increase efficiency in the delivery of health services. From the earliest days of mobile health (mHealth), the peer-reviewed literature has primarily focused on usability and feasibility of apps and has largely been published in the computer science literature [1,2]. Few, if any, studies focused on patient-level outcomes, especially in resource-poor settings in low- and middle-income countries [3]. Increasingly, mHealth has been embraced to strengthen delivery of maternal, neonatal, and child health (MNCH) services. There are numerous implementations globally; however, the evidence remains mixed.

Several systematic reviews highlight the lack of rigorous experimental and quasi-experimental trials; they also cite the lack of research measuring health outcomes as a key challenge to the advancement of the field of mHealth [4-9]. However, individual studies evaluating mHealth interventions have demonstrated an increased uptake of proven home- and facility-based practices and MNCH services. For example, an external evaluation of the *Chipatala Cha Pa Foni* project in Malawi, using a pre- and posttest design, demonstrated increased use of home- and facility-based maternal health practices and home-based child health practices among women exposed to the mHealth intervention (a toll-free case management hotline and automated and personalized mobile messages) [10]. Similarly, Lund et al used cluster randomized controlled trials to assess the *Wired Mothers* program in Zanzibar and demonstrated a significant increase of skilled delivery attendance and completion of four antenatal care visits among women receiving the mHealth intervention [11,12]. Although the observed improvements in proven practices may positively impact maternal and child health outcomes, no maternal or child health outcomes were directly measured in these studies.

Therefore, in this paper, we will share our experience and lessons learned from the evaluation of a large-scale mHealth voice message service implementation in India, where *real-world* health outcomes were assessed. We will also briefly highlight new possibilities for policy makers, implementers, and researchers to leverage electronic data to move toward *real-time* evaluation of outcomes from mHealth and other health interventions.

## The mMitra Program

The Mobile Alliance for Maternal Action (MAMA) was a 4-year public-private partnership focused on harnessing the power of mobile technology to send stage-based health messages to pregnant women and new mothers. There were country implementations of MAMA in Bangladesh and South Africa followed by India and Nigeria. Thus, the implementation and evaluation in India built on the program design and lessons from the earlier MAMA implementations and research studies.

In 2015, the MAMA partnered with the nonprofit organization Advancing Reduction in Mortality and Morbidity of Mothers, Children and Neonates (ARMMAN) to implement a mobile phone–based voice messaging service in the urban slums of Mumbai, India, called mMitra [13]. mMitra aimed to leverage mobile phone technology to support improvements in self-care among pregnant women living in urban slums and infant care.

A total of 145 voice messages were developed. The voice was female, and there were 2 languages available—Hindi and Marathi—the languages commonly spoken in the urban slums of Mumbai. The message development process was rigorous and involved BabyCenter, ARMMAN, and representatives from the Federation of Obstetric and Gynecologic Societies of India and the Indian Academy of Pediatrics. They were field tested with local health experts and community focus groups. The messages were delivered as a prerecorded phone call and covered the period from 6 weeks of pregnancy to an infant’s first birthday. A call-back service was integrated into the package for women to access within 2 days after the original call.

## Designing the Study

**Figure 1:**
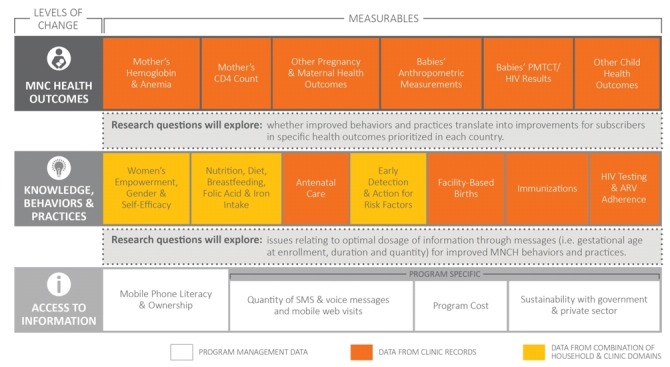
Mobile Alliance for Maternal Action theory of change and priority outcomes. ARV: antiretroviral; HIV: human immunodeficiency virus; MNC: maternal, newborn, and child; MNCH: maternal, newborn, and child health; PMTCT: prevention of mother to child transmission (of HIV); SMS: short message service.

A MAMA research agenda was developed to provide a standardized approach to evaluating the impact of the intervention in different countries [14]. A theory of change (see Figure 1) and targeted health outcomes for each of the countries were integrated into the agenda and helped inform the research design. All studies were designed to track and compare changes in key MNCH knowledge, attitudes, practices, and outcomes.

The underlying hypothesized pathway to change for mMitra was that if women receive educational messages that are interesting, easy to understand, and aligned with their physiological state during pregnancy and post delivery, then they would be motivated to take the needed self-care and seek the needed health services. There was an assumption that women would also have access to other sources of similar information, such as community health workers who enroll them for pregnancy care; health care providers who treat them at health centers; and mass media messages on radio, television, and posters.

As the MAMA studies assessed behavior, it was important to collect data from real-world sources, triangulate the data as a method of data validation, and juxtapose the self-reported data with objective clinical data (see Table 1).

**Table 1:** Prioritized health and behavior outcomes for Mobile Alliance for Maternal Action programs in Bangladesh, South Africa, and India with data sources.

Prioritized Health Outcomes		Bangladesh	South Africa	India
**MNCH^a^ Biomarkers of Health Outcomes**				
	Hemoglobin levels/ anemia in mothers	—^b^	—	Clinic records
	Babies’ anthropometric measurements	—	—	Clinic records
	CD4^c^ count from mothers with HIV	—	Clinic records	—
	WHO^d^ stage of mothers with HIV	—	Clinic records	—
	Tuberculosis status of mothers with HIV	—	Clinic records	—
	Babies HIV test result	—	Clinic records	—
**MNCH Behaviors, Practices, and Service Uptake**				
	Mother’s nutrition/diet/folic acid/iron tablets	Self-report	Self-report	Self-report
	Breast-feeding–exclusive and colostrum	Self-report	Self-report	Self-report
	Antenatal care	Self-report	Self-report	Self-report
	Gestational age at first ANC^e^	Self-report	Self-report	Self-report
	Facility-based births	Self-report	Self-report	Self-report
	Postnatal care	Self-report	Self-report	Self-report
	HIV counseling and testing	Self-report	Self-report	Self-report
	Antiretroviral therapy	Self-report	Self-report	Self-report
	Immunizations	Self-report	Self-report	Self-report
	Early detection and action for risk factors	Self-report	Self-report	Self-report
	Empowerment/gender/self-efficacy	Self-report	Self-report	Self-report

^a^MNHC: maternal, neonatal, and child health.

^b^Not applicable.

^c^CD4: Cluster of differentiation 4.

^d^WHO: World Health Organization.

^e^ANC: antenatal care.

**Figure 2:**
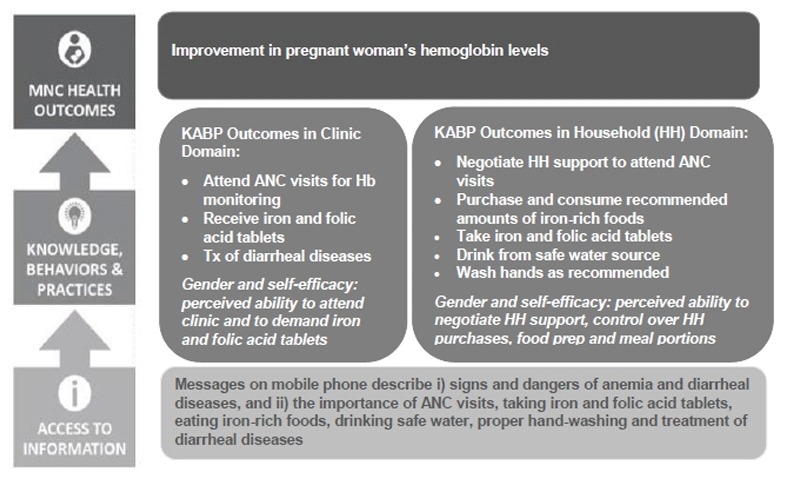
Pathway to change for hemoglobin levels in mMitra. ANC: antenatal care; Hb: hemoglobin; HH: household; KABP: knowledge, attitudes, behavior, and practices; MNC: maternal, newborn, and child.

The lack of objective clinical data in the Bangladesh evaluation led to the collection of retrospective clinical data in South Africa [15,16] and an attempt to use maternal and child health record data from the public health system in India. The measurement of physiologic biomarkers, reflecting health outcomes, were included from the start of the implementation in India (see Figure 2). The biomarkers selected were maternal hemoglobin levels and the infant’s birth weight. These reflected the country’s public health priorities in particular based on the India District Level Health Survey conducted in 2013, which documented that 68.5% pregnant women among the urban poor in Maharashtra are anemic, babies with normal birthweight were 87%, and babies being normal or mildly undernourished at year 1 were 63%.

Given the evolving technology landscape (ie, more affordable technologies and emergence of new technologies), evaluation studies are often not relevant by the time full-scale implementation occurs [17]. There is also an ongoing debate on the appropriateness of randomized controlled trials as the gold standard for mHealth evaluation [5,18,19]. In addition, evaluating behavior change adds complexity, as it is difficult to attribute improvements in health behaviors to the intervention alone. To aid in the study design, and afterwards evaluation and reporting, we used the following resources, respectively:

- Whittaker et al’s detailed process on rigorous mHealth experimental evaluation approaches and types of measures that should be included [17].
- Roess’ publication that outlines process evaluation and implementation science approaches that can be used to measure reach, fidelity of use, and the *dose* of an intervention [20].
- The mHealth evidence reporting and assessment checklist, which is often used to guide reporting on the effectiveness of digital health [21,22].

We ultimately decided to use a pseudo-randomized controlled trial design to assess the impact of mMitra on the desired outcomes [23]. The study design was chosen for its experimental nature, and it built on the lessons learnt from the prior MAMA implementations and evaluations. Importantly, the study design followed women, by trimester, allowing for a dose-response assessment.

All women interested in participating in the study and implementation provided written informed consent. The study was approved by the Foundation for Research in Health System’s institutional review board (under protocol no. HHS00009235) and registered with ISRCTN (registration no. 88968111). Specially designed questionnaires were administered to women in the control and intervention groups at 3 time points: at enrollment in the study while the women were pregnant (baseline), after delivery, and when their baby reached 1 year of age. The surveys were conducted in-person with consent in Hindi and Marathi with the enumerator using an Android-based data collection platform. At the same in-person encounters, data were abstracted, with consent, from the woman’s Maternal and Child Health (MCH) card. The MCH card is issued to women by hospitals where they register for prenatal care. It serves as their health record, and they are instructed to bring the card with them on every visit for staff to update. Women are incentivized to keep their cards as it determines their eligibility to receive government compensation after delivery. Of the women interviewed post delivery 1040/1113 (93.44%) in intervention and 381/402 (94.8%) in control group showed their MCH card to the enumerators.

## Lessons Learned

Several key lessons were learned through this process. Major insights were incorporated into the study design based on the previous evidence and approach to research of other mobile messaging programs, the literature related to mHealth research, and the cumulative experience in research across the initial 2 MAMA country programs. This led to the adoption of a prospective pseudo-randomized controlled trial that proactively measured dose response based on the duration of exposure to messages by trimester and integrated objective clinical data with self-reported information.

As we had assumed that women would be exposed to external sources of information that would influence their behavior and practices, we prioritized real-world study designs over pure experimental designs. This pragmatic approach allowed us to have a comparison group and ensure that we would appropriately attribute any potential impact of the intervention on behavior and health outcomes, if any. We were also able to ensure that the research aligned with India’s health priorities by ensuring the study’s health outcomes matched the country’s priorities.

The triangulation of the data helped advance linkages between the intervention and clinical outcomes. Through this process, we learned that even though the health record (MCH card) seemed like a reliable data source because of government incentives, it did not preclude missing and poor-quality data. This was particularly true for maternal health data but less so for child health data. Only 24% of MCH cards in the control group and 34% of cards in the intervention group had entries for maternal hemoglobin—a key maternal health outcome of interest; whereas 65% of cards in both groups had anthropometric child health data that could be used to evaluate key child health outcomes. However, the data from the MCH cards were insufficient, and findings detected from the subsample of MCH cards with complete data were not statistically significant. This impacted our ability to use the data from the MCH cards to assess clinical health outcomes, but it provided an authentic, real-world perspective. It is acknowledged that the issue of missing data may vary by data element as well as by data source as well as use case and context.

From the outset of the study, we kept track of the duration of exposure women had to the messages. This was important to track to determine if a dose response was present. However, we also recognized that greater confidence could be placed in the message exposure findings by having both self-reported and electronic data sources. Subsequently, this brought attention to a future consideration—the inclusion of *data exhaust* generated by the messaging platform and linking this type of message data with research data for a direct one-to-one correlation between messages and effects. Data exhaust are trails of data left by users. Examples of data exhaust include the success rate of phone calls being delivered and accessed, the number of missed calls by message type and timing, and the numbers of call-backs accessed. Data exhaust can be used to aid with determining message access and user trends; it can be useful for ensuring completeness and accuracy of research data without additional human effort for data quality assessments. They are also generated in *real-time*, which can help shift programming toward more timely decision making.

With the increase in the digitization and use of decision-support and community-case management tools by frontline health workers, there are opportunities to further leverage existing data collection processes to validate and supplement self-reported and health record data. Furthermore, future assessments on the nature of the missing data may provide useful insights into how it can be addressed through statistical methods or through efforts made upstream to the data collection (eg, frontline health workers). As research moves toward real-world evidence and data generated through digital systems, it is important for the initial design to abide by data ownership and governance regulations, including mechanisms for obtaining consent for the use of routine health data to be used for research purposes as well as promoting data completeness and data quality. A rigorous focus on these aspects will allow evaluations to harness data more effectively and better lend themselves to high-quality research efforts that generate sound evidence.

## Conclusions

The MAMA research experience, especially that of mMitra in India, provides important considerations for how to approach health outcomes research in evaluating the impact of an mHealth intervention. Having a clear understanding of the context and local health priorities is important to ensure that the research agenda is in alignment with local and national priorities and, thus, can have greater relevance to policy makers, program implementers, and beneficiaries. The inclusion of biomarkers and use of clinical records were an attempt to complement self-reported information on health outcomes. These opportunities are increasing as more health systems increase adoption of electronic medical records and shared health records. However, as our experience highlights, real-world data has its limitations—particularly in the areas of data completeness and quality. There is also an opportunity to use the data exhaust and meta-data generated from digital tools as an additional data source. Further study is recommended to assess the impact of missing data and the quality and viability of these types of records for future use in health outcomes–focused mHealth research. As more technology is adopted by health systems, the ability to move toward real-world evidence in the evaluation of digital health interventions and health interventions alike will increase—but great care and adoption of ethical practices will be required in the way that data are captured, structured, managed, analyzed, and shared.

## Abbreviations

ARMMAN: Advancing Reduction in Mortality and Morbidity of Mothers, Children and Neonates
MAMA: Mobile Alliance for Maternal Action
MCH: maternal and child health
mHealth: mobile health
MNCH: maternal, neonatal, and child health
